# Inhibition of BMP and of TGFβ receptors downregulates expression of XIAP and TAK1 leading to lung cancer cell death

**DOI:** 10.1186/s12943-016-0511-9

**Published:** 2016-04-06

**Authors:** Dave J. Augeri, Elaine Langenfeld, Monica Castle, John A. Gilleran, John Langenfeld

**Affiliations:** Rutgers Translational Sciences, Department of Medicinal Chemistry, School of Pharmacy, New Brunswick, NJ USA; Division of Surgical Oncology, Rutgers Cancer Institute of New Jersey, MEB 536, One Robert Wood Johnson Place, P.O. Box 19, New Brunswick, NJ 08903-0019 USA

**Keywords:** BMP, TGFβ, XIAP, Id1, TAK1, Cell death

## Abstract

**Background:**

Bone morphogenetic proteins (BMP) are embryonic proteins that are part of the transforming growth factor (TGFβ) superfamily, which are aberrantly expressed in many carcinomas. Inhibition of BMP receptors with small molecule inhibitors decreases growth and induces death of lung cancer cells, which involves the downregulation of Id1 and Id3 by a Smad dependent mechanism. Developmentally, BMP and TGFβ signaling utilizes Smad-1/5 independent mechanisms to stabilize the expression of X-linked inhibitor of apoptosis protein (XIAP) and activate TGFβ activated kinase 1 (TAK1), which are known to be potent inhibitors of apoptosis. The role of BMP signaling in regulating XIAP and TAK1 in cancer cells is poorly understood. Furthermore, the interaction between the BMP and TGFβ signaling cascades in regulating the activation of TAK1 in cancer cells has not been elucidated.

**Methods:**

Feedback regulation between the BMP and TGFβ signaling pathways and their regulation of XIAP, TAK1, and Id1 were examined in lung cancer cells utilizing siRNA and inhibitors targeting BMP type I receptors, inhibitors of BMP and TGFβ type I receptors, and an inhibitor of BMP and TGFβ type I and type II receptors.

**Results:**

We show that upon inhibition of BMP signaling in lung cancer cells, the TGFβ signaling cascade is activated. Both the BMP and TGFβ pathways activate TAK1, which then increases the expression of Id1. Inhibition of TGFβ signaling increased Id1 expression except when BMP signaling is suppressed, which then causes a dose-related decrease in the expression of Id1. Inhibition of both BMP and TGFβ signaling enhances the downregulation of TAK1. Our data also suggests that the blockade of the BMP type II receptor enhances the downregulation XIAP, which is important in decreasing the activity of TAK1. Knockdown studies demonstrate that both XIAP and TAK1 regulate the survival of lung cancer cells.

**Conclusions:**

This paper highlights that targeting the BMP and TGFβ type I and type II receptors causes a downregulation of XIAP, TAK1, and Id1 leading to cell death of lung cancer cells. Small molecule inhibitors targeting the BMP and TGFβ receptors represents a potential novel means to treat cancer patients.

**Electronic supplementary material:**

The online version of this article (doi:10.1186/s12943-016-0511-9) contains supplementary material, which is available to authorized users.

## Background

Lung cancer is the leading cause of cancer deaths in the United States. An estimated 185,000 people are expected to die this year in the United States from lung cancer. More patients succumb to lung cancer than breast, colon, and prostate cancer combined. Despite advances in medical care, 85 % of patients diagnosed with lung cancer will die from their disease. It is clear from these dismal statistics that novel therapeutic targets and treatment strategies are needed for the treatment of lung cancer.

Bone Morphogenetic proteins (BMP) are members of the Transforming Growth Factor superfamily (TGFβ). BMP2 and BMP4 are phytogenetically conserved morphogens required for embryonic development across species from insects to humans [1, 2]. Upon completion of lung morphogenesis BMP signaling is barely detectable in normal adult lung tissue [2]. BMP signaling is reactivated in lung injury and non-small cell lung (NSCLC) and small cell carcinomas [3, 4]. BMP2 expression is highly over-expressed in 98 % of NSCLC compared to normal lung tissue and benign lung tumors [3]. The activation of BMP signaling has been implicated in the tumorigenesis of lung and many other carcinomas. BMP2 induces tumor angiogenesis [5], stimulates the migration [4] and metastasis of cancer cells [6, 7], and its expression is associated with a worse prognosis [8].

Approximately 20 BMP ligands have been identified and categorized into several subclasses. BMPs signal through transmembrane serine/threonine kinases composed of type I and type II receptors. The type I receptors are ALK1, ALK2 (ActR-1), ALK3 (BMPR-IA), and ALK6 (BMPR-IB) [9]. The type II receptors are BMPR-II and activin type II receptors ActR-II and AcR-IIB [9]. Each BMP receptor can be activated by several different BMP ligands [9]. Binding of the BMP ligand to the type I receptor leads to phosphorylation by the constitutively active type II receptor. This receptor complex then phosphorylates Smad-1/5 [10] and activates the transcription of downstream target genes including inhibitor of differentiation proteins (Id1, Id2, and Id3) through BMP response elements on their promoter [11–15].

Recent studies have demonstrated that the BMP signaling cascade promotes growth and survival of lung cancer cells [16]. Downregulation of the type I BMP receptors with siRNA or small molecule inhibitors (DMH2, DMH1) in lung cancer cells caused growth inhibition and cell death, which is associated with a down-regulation of Id1 and Id3 [16]. Knockdown of either Id1 or Id3 also suppressed growth and induced cell death [16]. Forced expression of Id3 attenuated growth suppression and cell death caused by BMP receptor inhibitors [16]. These studies demonstrate that the BMP signaling cascade promotes tumorigenesis that involves a Smad 1/5 dependent regulation of Id1-Id3. This report also highlighted that small molecule inhibitors of the BMP receptors can be used to interrogate the BMP signaling cascade in cancer cells and represents a potential therapeutic strategy for the treatment of lung and other cancers.

**Scheme 1:**
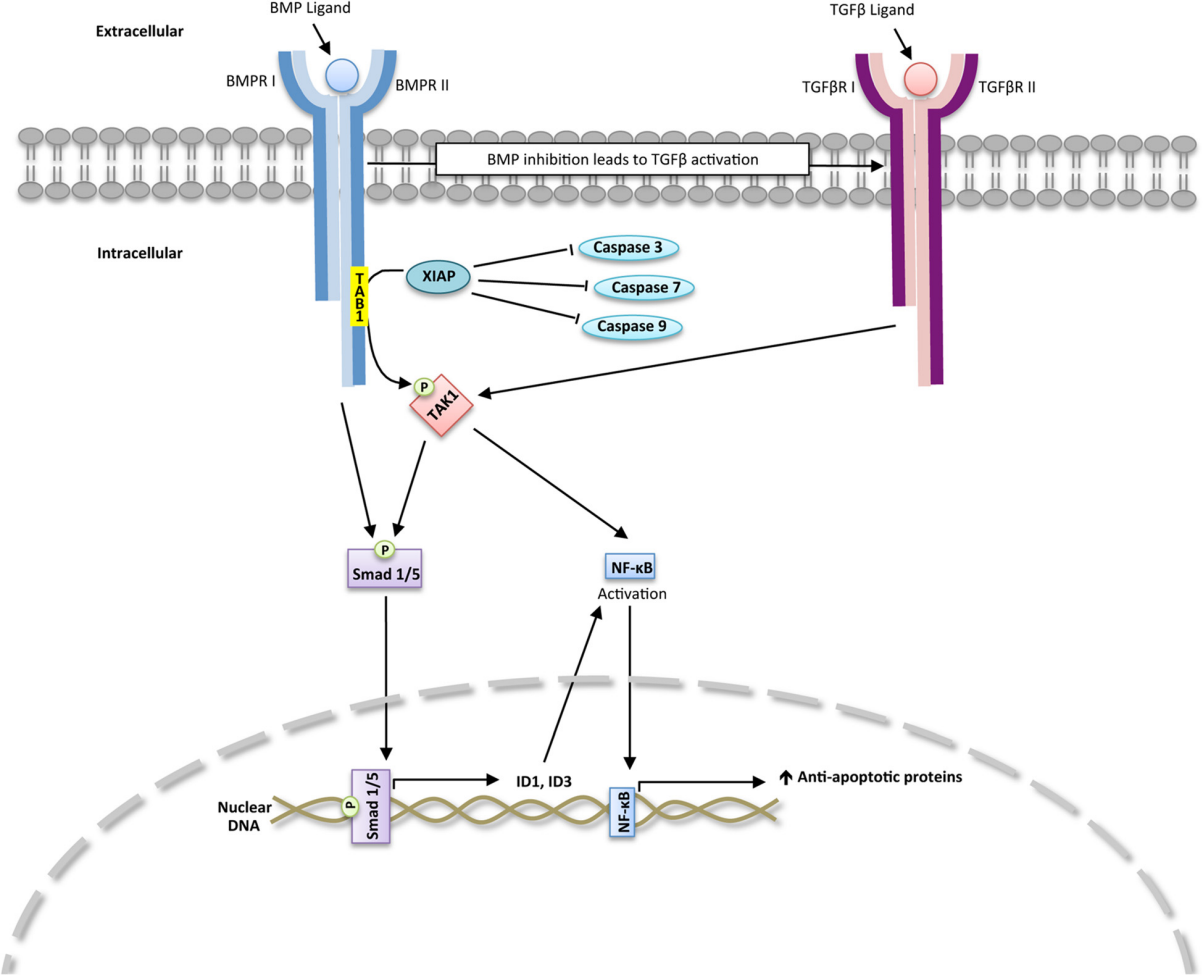
BMP pathway

The regulation of Id proteins by the BMP signaling cascade has important therapeutic implications. There are numerous reports demonstrating the importance of Id proteins promoting tumorigenisis in many types of cancers. Expression of Id proteins by immortalize cells stimulate tumor invasion and metastasis and are essential for tumor angiogenesis. The expression of Id1 is necessary for Ras induced tumor formation by inhibiting senescence [17]. There are limited data demonstrating other signaling pathways that are direct transcriptional regulators of Id proteins. Scr is reported to enhance the transcription of Id1 by binding the BMP transcriptions factors Smad-1/5, which promotes its translocation into the nucleus and activation of the Id1 promoter [18]. One report suggested mitogen-activated protein kinases (MEK-1/2) may promote the transcription of the Id1 promoter through its regulation of the early growth response protein (Egr-1) [19]. The TGFβ signaling cascade acts predominately to decrease the expression of Id1 [20, 21]. In some cells, TGFβ signaling has been shown to increase the transcription of Id1 [22]. During development, the BMP signaling cascade suppresses (MEK-1/2) activity to promote self-renewal of mouse embryonic stem cells [23]. The BMP pathway (Scheme 1) has also been shown to regulate the expression of Src through its interaction with the BMPRII [24]. These studies highlight the potential for feedback regulation between the BMP, Scr, TGFβ, and/or MEK-1/2 effecting the transcription of Id proteins thereby affecting the potency of BMP receptor inhibitors.

Through evolutionary conserved pathways, the BMP signaling cascade also regulates embryonic development through Smad-1/5 independent mechanisms. The activation of TGFβ activated kinase 1 (TAK1) by the BMP signaling cascade is required for ventralization of embryos [25]. The BMP signaling cascade regulates TAK1 by increasing the expression of x-linked inhibitor of apoptosis protein (XIAP) through its binding to the cytoplasmic domains of the type I and type II BMP receptors [26]. XIAP binds TAK1-binding protein (TAB1) that recruits TAK1 leading to its activation [25]. Both the TGFβ and BMP signaling cascades induce the activation of TAK1 and stabilize the expression of XIAP. TAK1 can induce a feed-forward activation of BMP signaling by phosphorylating Smad 1/5 [27]. TAK1 and XIAP are potent inhibitors of apoptotic cell death in cancer cells [28, 29]. The regulation of XIAP and TAK1 by the BMP signaling cascade in cancer cells is poorly elucidated. It is also not known whether the expression of XIAP and activity of TAK1 is regulated by BMP receptor inhibitors. The one report examining BMP regulation of XIAP in cancer cells showed that BMPRII in osteosarcoma cell lines stabilized the expression of XIAP [30]. Furthermore, it is not known whether the BMP and TGFβ signaling cascades cooperate to regulate XIAP and TAK1 in cancer cells.

Using inhibitors blocking the activity of specific type I and type II BMP and TGFβ receptors together with RNAi knockdowns, we examined the mechanisms by which the blockade of the BMP receptors regulates survival of lung cancer cells. We found that suppression of BMP signaling results in feedback activation of the TGFβ signaling, which gives rise to the activation of TAK1. BMPRII regulates the expression of XIAP in lung cancer cells, which activates TAK1. We demonstrate that a small molecule inhibitor of BMP receptors downregulates the expression of XIAP and TAK1 in lung cancer cells. We provide evidence supporting that an inhibitor targeting the BMP and TGFβ type I and type II receptors will result in the greatest downregulation of Id1, TAK1, and XIAP and induction of apoptotic cell death of lung cancer cells. This paper provides a mechanistic rational directing further drug development to target the BMP/TGFβ signaling cascades for the treatment of cancer.

## Results

### DMH2 inhibits the expression of Id1 and growth of different types of lung cancer cells

To better understand the role of BMP inhibitors to treat lung cancers, the potency of the BMP receptor inhibitor, DMH2 [16, 31], to decrease the expression of Id1 and its effect on cell growth was examined in a panel of lung cancer cell lines of different cell types and mutations commonly found in lung cancer. The lung cancer cell lines examined consisted of a squamous carcinoma (H157), adenocarcinoma (A549), poorly differentiated carcinoma (H1299), low-grade (H727) and high-grade (U1752) neuroendocrine carcinomas. The mutations included a p53 deletion (H1299), activating K-Ras mutations (A549, H157, H727), and PNET deletion (H157). DMH2 decreased the expression of Id1 (Additional file 1: Figure S1A) and inhibited growth in all of the cell lines (Additional file 1: Figure S1B). These studies suggest that inhibition of BMP signaling may have significant anti-tumor effects across a broad range of lung carcinomas.

### BMP Inhibitors increase expression of Id1 in tumor xenografts

**Fig. 1:**
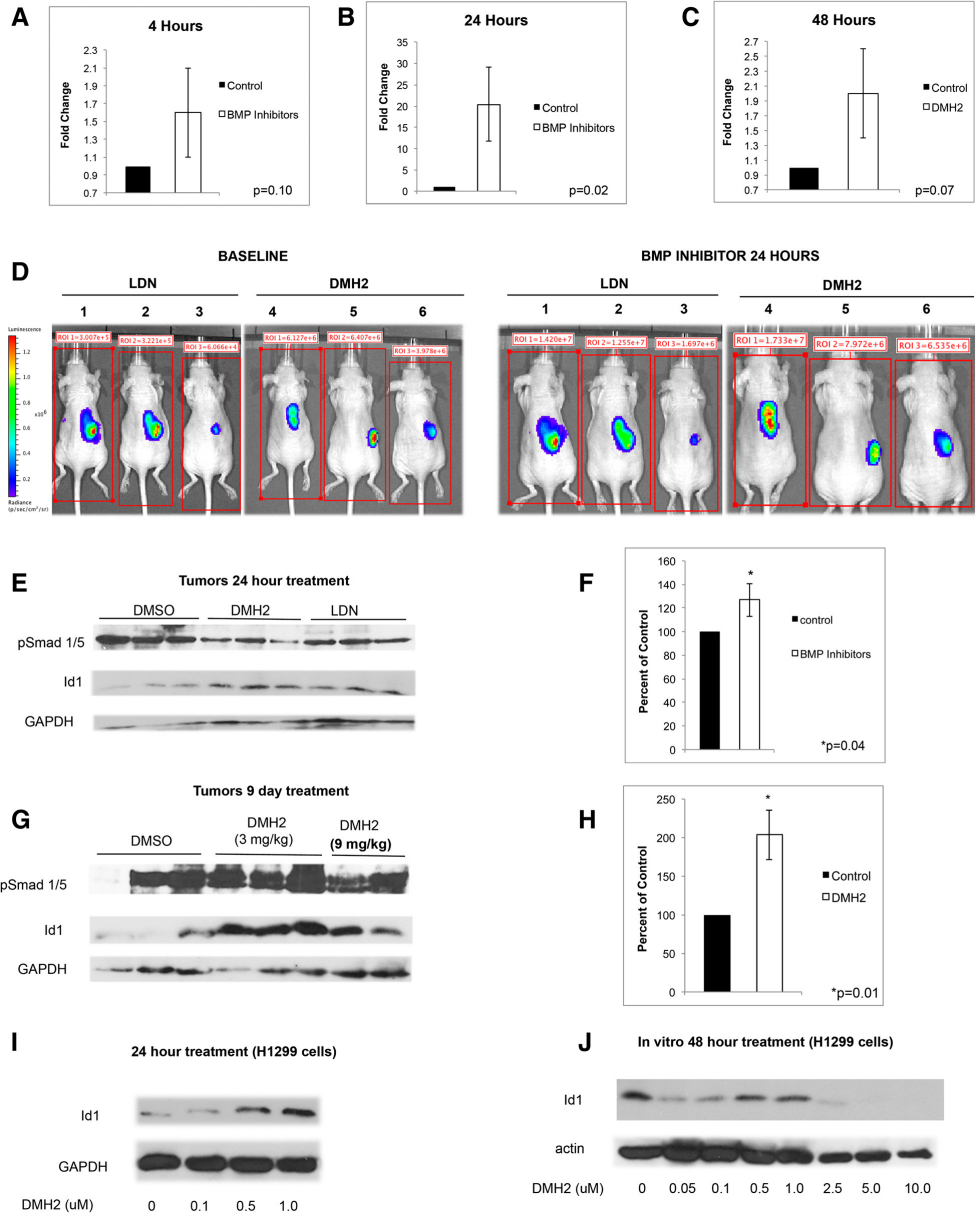
DMH2 causes an increase in Id1 expression in tumor xenografts and in vitro at low concentrations. **a** Mice with established tumors from H1299 Id-luc cells were treated with DMSO or 3 mg/kg of DMH2 or 3 mg/kg LDN and after 4 h tumor luminescence determined and compared to baseline (*n* = 3 for each group). (**b**) Two days later mice were treated with DMSO or 3 mg/kg of DMH2 or 3 mg/kg LDN every 8 h for 24 h and tumor luminescence determined and compared to baseline. (**c**) In a separate experiment, established tumors were treated with DMSO (*n* = 3) or 3 mg/kg of DMH2 (*n* = 5) twice daily for 48 h and luminescence was compared to baseline. (**d**) Bioluminescence images of H1299 Id1-luc tumors before and after treatment with BMP inhibitors for 24 h. Data depicted as fold increase in tumor luminescence of mice treated with inhibitors compared to baseline. (**e**) Western blot analysis of tumor xenografts from mice treated with DMSO or 3 mg/kg of DMH2 or 3 mg/kg LDN every 8 h for 24 h and (**f**) the mean optical density readings of Id1 from the corresponding Western blot was normalized to GAPDH and presented as percent of control. (**g**) Western blot analysis of xenografts from mice treated with twice-daily injection of DMH2 for 9 days and (**h**) the mean optical density readings of Id1 from the corresponding Western blot was normalized to GAPDH and presented as percent of control. (**i**, **j**) Western blot analysis of H1299 cells in cell culture treated with increasing doses of DMH2 for 24 (*n* = 2) (**i**) and (**j**) 48 h (*n* = 4)

The effect of the BMP receptor inhibitors DMH2 and LDN-193189 (LDN) [32] to regulate Id1 expression in tumor xenografts was examined. Established xenografts from H1299 cells stably expressing an Id1 promoter regulating luciferase were treated with DMH2 or LDN and tumor luminescence was measured before and after treatment. Tumor luminescence was increased at 4 h, 24 h, and 48 h following treatment compared to baseline with the greatest effect seen at 24 h (Fig. 1a–d). In total, 13 of 17 mice treated with a BMP inhibitor showed an increase in Id1 promoter activity in the tumor (Fig. 1a–d). Immunoblot analysis demonstrated a 27 % increase in ID1 expression in xenografts treated with BMP inhibitors for 24 h as compared to controls (Fig. 1e and f). At 9 days following treatment with DMH2 there was a 103 % increase in the expression of Id1 protein compared to controls (Fig. 1g and h). We examined in vitro the dose responsive regulation of Id1 in H1299 and A549 cells following treatment with DMH2 for 24 and 48 h. These studies showed that low doses of DMH2 as low as 0.5 μM can cause an increase in the expression of Id1 followed by suppression of Id1 expression at higher concentrations (Fig. 1i and j, Additional file 2: Figure S2). In the study for the H1299 cells, we found a decrease in Id1 expression followed by an increase and complete suppression at higher doses. These studies are consistent with BMP inhibition leading to feedback activation of Id1 expression with lower concentration of BMP inhibitor.

### Pharmacokinetics of DMH2

We next examined the bioavailability of DMH2. The percent of DMH2 binding plasma proteins in human and mouse plasma is 98.6 % +/−0.7 and 98.2 % +/−1.5 respectively. DMH2 exhibited moderate to high systemic plasma clearance (68.45 mL/min/kg) with elimination half-life of 0.95 h (Additional file 2: Figure S2A). LDN is reported to have an elimination half-life of 90 min in mice [32]. The IC50 of DMH2 for the BMP type I receptor Alk2 is reported to be 42.8 nM [31]. Therefore, the free fraction of unbound DMH2 above its IC50 for Alk2 is less than 25 min (Additional file 3: Figure S3B). These pharmacokinetic studies suggest that DMH2 would provide only weak inhibition of BMP signaling in tumor xenografts. These studies suggested that DMH2 might be a weak inhibitor in vivo, which allows other signaling pathways to regulate the transcription of Id1.

### MEK-1/2 and Src do not cause feed-back activation of Id1

To assess potential activated feedback loops following inhibition of BMP signaling, we examined the pathways reported to regulate the transcription of Id1. Src was not activated in tumors treated with BMP inhibitors for 24 h or 9 days. Although inconsistent, some of the tumors treated with BMP receptor inhibitors had an increase in the expression of phosphorylated MEK-1/2 (Additional file 3: Figure S3A and B). In vitro, DMH2 caused an increase in the expression of phosphorylated MEK-1/2 in H1299 (Additional file 3: Figure S3C) and A549 cells (Additional file 4: Figure S4D). We examined whether Egr-1, the transcriptional regulator of Id1 induced by pMEK-1/2, was regulated following BMP inhibition. We found no change in the expression of Egr-1 with the activation of MEK-1/2, suggesting MEK-1/2 did not regulate Egr-1 in the H1299 cells (Additional file 4: Figure S4C). The activation of MEK-1/2 corresponded with a decrease in the expression of Id1 (Additional file 4: Figure S4D and Additional file 1: Figure S1J) and not an increase as would be expected if it where activating the transcription of Id1. The specific MEK-1/2 inhibitor PD0325901 (PD) [33] did not regulate the promoter activity of Id1 as demonstrated in the Id-1 luciferase reporter assay in H1299 cells (Additional file 4: Figure S4E). Furthermore, PD when combined with DMH2 had no additional effects on growth inhibition of H1299 and A549 cells compared to DMH2 alone (Additional file 4: Figure S4F-G). These studies support that Scr and MEK-1/2/Egr-1 signaling pathways were not the mechanism causing the increase in Id1 expression following low levels of inhibition of BMP signaling.

### Suppression of BMP signaling results in activation of TAK1 and TGFβ

**Fig. 2:**
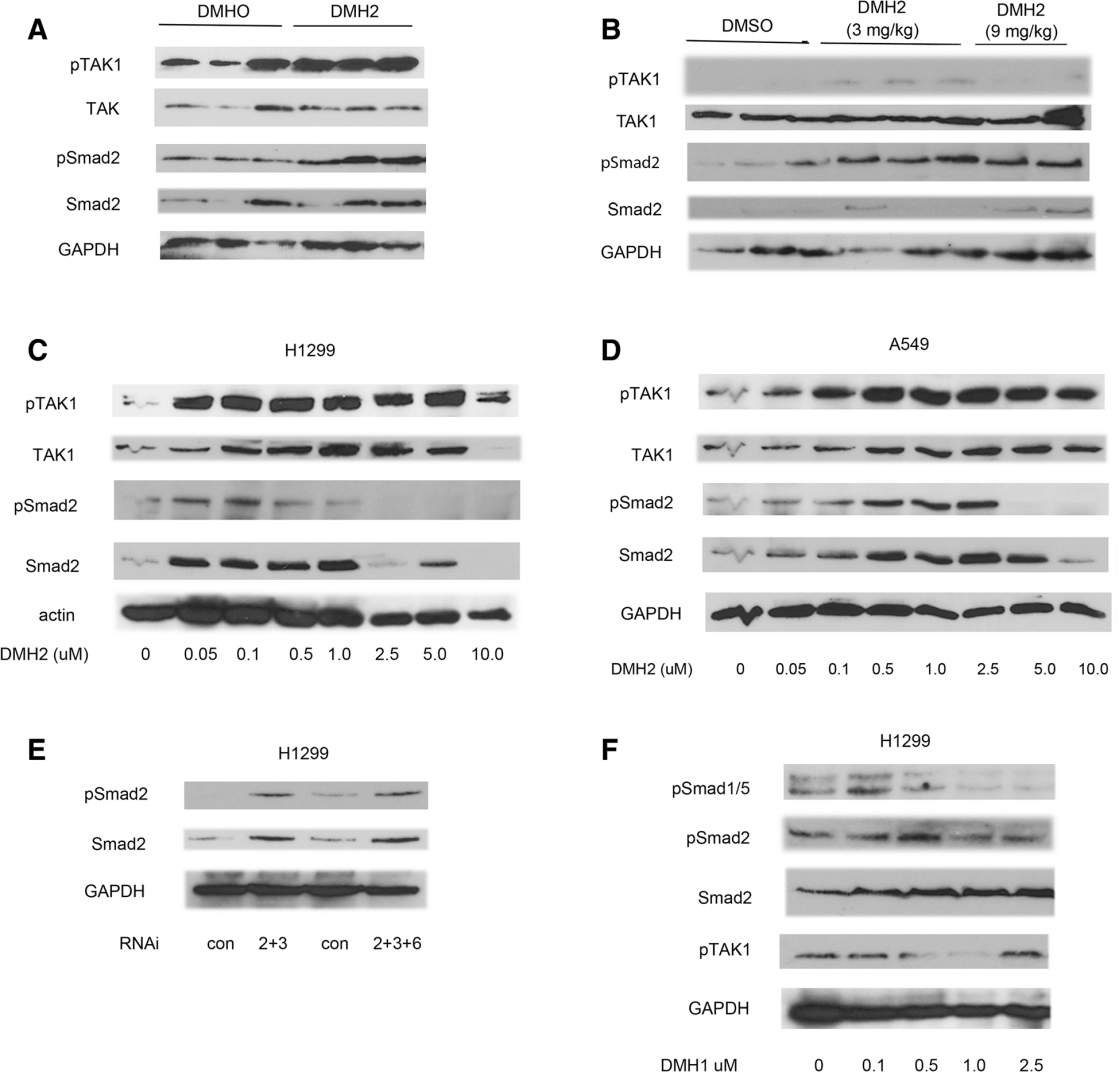
Inhibition of BMP signaling activates TAK1 and Smad2. **a**, **b** Western Blot analysis of H1299 tumor xenografts treated with DMH2 for 24 h and 9 days showing inhibitors increase expression of pSmad2 and pTAK1. (**c**, **d**) Western blot analysis of H1299 and A549 cells treated with increasing doses of DMH2 in vitro for 48 h showing an increase followed by a decrease in expression pTAK1 and pSmad2 (*n* = 4). (**e**) Western blot of knockdown of alk2 and alk3 or alk2, alk3, and alk6 showing an increase in pSmad2 and Smad2 expression (*n* = 2). (**f**) Western blot of cells treated with DMH1 in vitro for 48 h (*n* = 3)

Since both the BMP and TGFβ signaling cascades regulate TAK1, which has been shown to cause a feed-forward activation of BMP signaling in cartilage progenitor cells [34], we next examined the activation of TAK1 and TGFβ following the suppression of BMP signaling. Tumor xenografts treated with DMH2 for 24 h and 9 days showed activation of TAK1 and TGFβ signaling as demonstrated by an increase in phosphorylated TAK1 and Smad2 respectively (Fig. 2a and b). In vitro, DMH2 caused an increase in phosphorylation of TAK1and Smad2 at lower doses, which tapered off at higher doses (Fig. 2c and d). The decrease in activity of Smad2 corresponded with a significant decrease in the expression of Id1 in both H1299 and A549 cells (Fig. 1j and Additional file 3: Figure S3D). The unphosphorylated Smad2 was regulated in a similar fashion except that its downregulation occurred at a higher concentration than pSmad2 (Fig. 2c and d). Knockdown of the BMP type I receptors alk2 and alk3 as well as alk2, alk3, and alk6 caused an increase in expression of Smad2 and pSmad2 (Fig. 2e). DMH1, an inhibitor specific for BMP type I receptors with no activity for TGFβ receptors [31], also caused the activation of Smad2 and TAK1 (Fig. 2f). Unlike DMH2 that inhibits the TGFβ type I receptor Alk5 [31], DMH1 did not decrease the expression of Smad2 or pTAK at higher doses. These studies show that suppression of BMP signaling results in a feed-back increase in the expression and activation of Smad2 and TAK1. These studies also demonstrated a functional difference between the BMP inhibitors DMH1 and DMH2.

### DMH2 is more potent than DMH1 and has a different IC50 profile

We previously reported that DMH2 causes a greater decrease in the expression of Id1 protein and cell growth in comparison to DMH1 [16]. To better understand differences between DMH1 and DMH2, we determined the IC50 of DMH2 for all the BMP type I receptors, Alk5, as well as BMPRII and TGFβ type II receptors and compared it to the reported literature for DMH1 and LDN (Table 1) [35]. Both DMH1 and DMH2 inhibited Alk2 and Alk3 at nanomolar concentrations. DMH2 inhibited Alk5 at an IC50 of 1690 nM, which would account for the decrease in pSmad2 expression at 2.5 μM (Fig. 2c and d). Unique to DMH2 was the inhibition of BMPRII, which does not occur with DMH1 or LDN (Table 1). DMH2 causes significantly greater suppression of Id1 promoter activity at 1.0 μM and 2.5 μM in the H1299 cells in comparison to DMH1 (Additional file 5: Figure S5). We also found that DMH2 induces significantly greater growth suppression and cell death of H1299 cells compared to DMH1 and LDN (Additional files: Figure S6 and Figure S5). These data suggest that the inhibition of both the BMP and TGFβ signaling cascades may enhance the downregulation of Id1 leading to greater growth inhibition and cell death.

**Table 1:** IC50

Half maximal inhibitory concentration (IC50)							
	Alk2/ACVRR1 (nM)	Alk3/BMPR1A (nM)	Alk6/BMPRII (nM)	BMPRII (nM)	Alk5/TGFbetaR1 (nM)	TGFbetaR2 (nM)	
DMH2	23	92	43	7,950	1,690	193	
DMH1	108	5	–	–	–	–	(35) (35)
LDN	5	20	3	–	185	151	

Inhibition of kinase activity of BMP and TGF beta receptors was determined for DMH2 and compared to DMH1 and LDN

### TAK1 activates BMP signaling

**Fig. 3:**
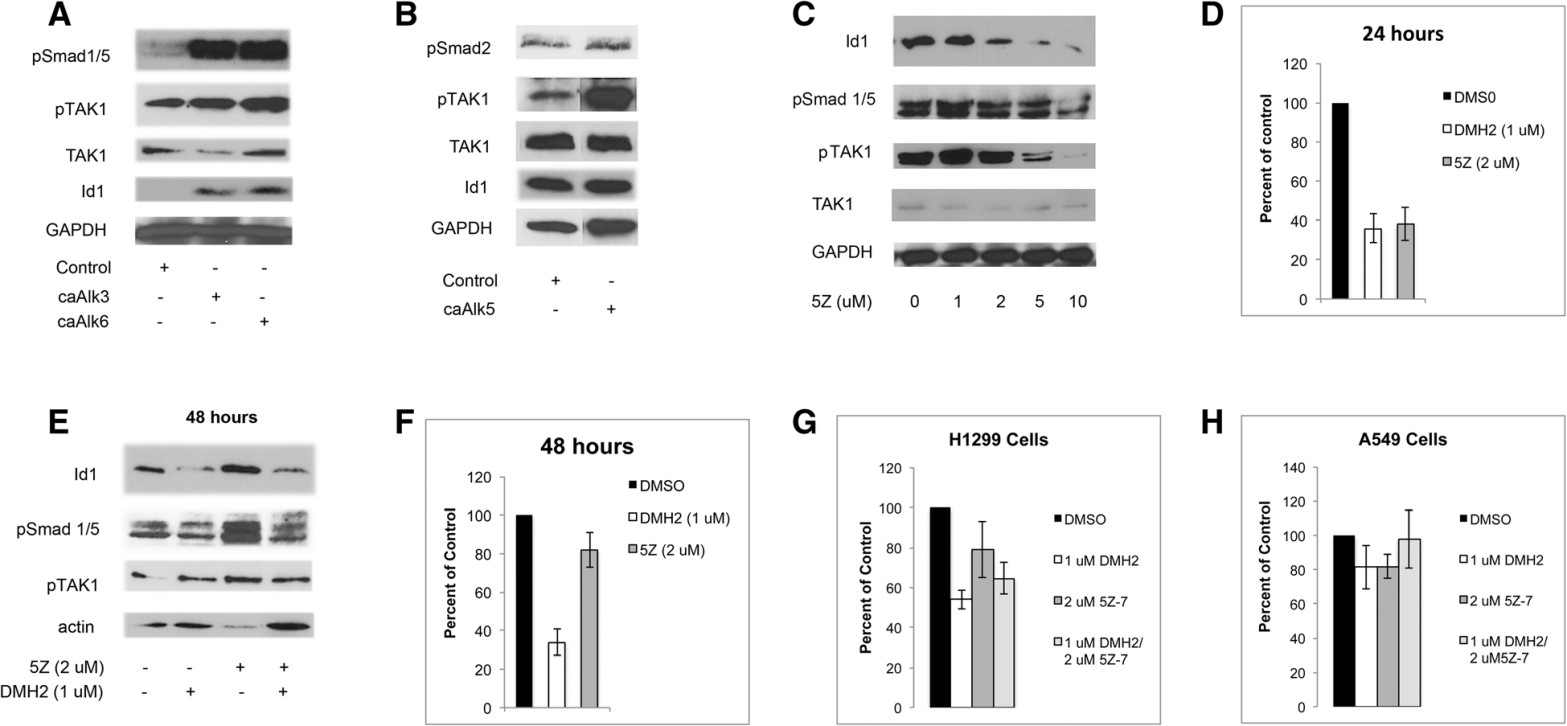
BMP and TGFβ signaling activates TAK1 which stimulates BMP signaling. **a**, **b** Western blot analysis showing that transient expression of constitutively active alk3, alk6, or alk5 increases expression of pTAK1 (*n* = 3). (**c**-**f**) TAK1 antagonist 5Z-7-oxozeaenol (5z) decreases BMP signaling after 24 h but increases it at 48 h (*n* = 3). (**c**) Western blot analysis and (**d**) Id1-luciferase assay of H1299 cells treated with 5z for 24 h. (**e**) Western blot analysis and (**f**) Id1-luciferase assay of H1299 cells treated with 5z and DMH2 for 48 h. (**g**, **h**) H1299 cells were treated with DMH2 or 5Z-7-oxozeaenol (5Z-7) alone, and in combination for 7 days and the number of live cells determined. Data in growth assays were depicted as the percent of the DMSO control. All studies were performed at least 3 times

We next examined whether TAK1 causes a feed-forward activation of BMP signaling in lung cancer cells and whether both the BMP and TGFβ signaling pathways activated TAK1. Transient expression of constitutively active BMP and TGFβ type I receptors caused the activation of TAK1 and Id1 expression in the H1299 cells (Fig. 3a and b), confirming their role in activating TAK1 in our lung cancer cells. The irreversible TAK1 inhibitor, 5Z-7-oxozeaenol (5Z) [36], causes a dose-related decrease in the expression of pSmad 1/5, Id1, and pTAK1 after 24 h (Fig. 3c). After 24 h, 2 μM of 5Z also suppressed Id1 promoter activity (Fig. 3d). Interestingly, after 48 h 5Z had the opposite effect, now increasing the expression of Id1, pSmad 1/5, and pTAK1, and no longer suppressed the Id1 promoter (Fig. 3e and f), demonstrating that 5Z induces feedback activation of its intended target. Furthermore, the combination of 5Z and DMH2 also did not enhance growth suppression (Fig. 3f–h). These data suggest that activated TAK1 stimulates Smad 1/5-Id1 signaling through a feed-forward mechanism in lung cancer cells.

### TGFβ signaling increases Id1 expression by activating TAK

**Fig. 4:**
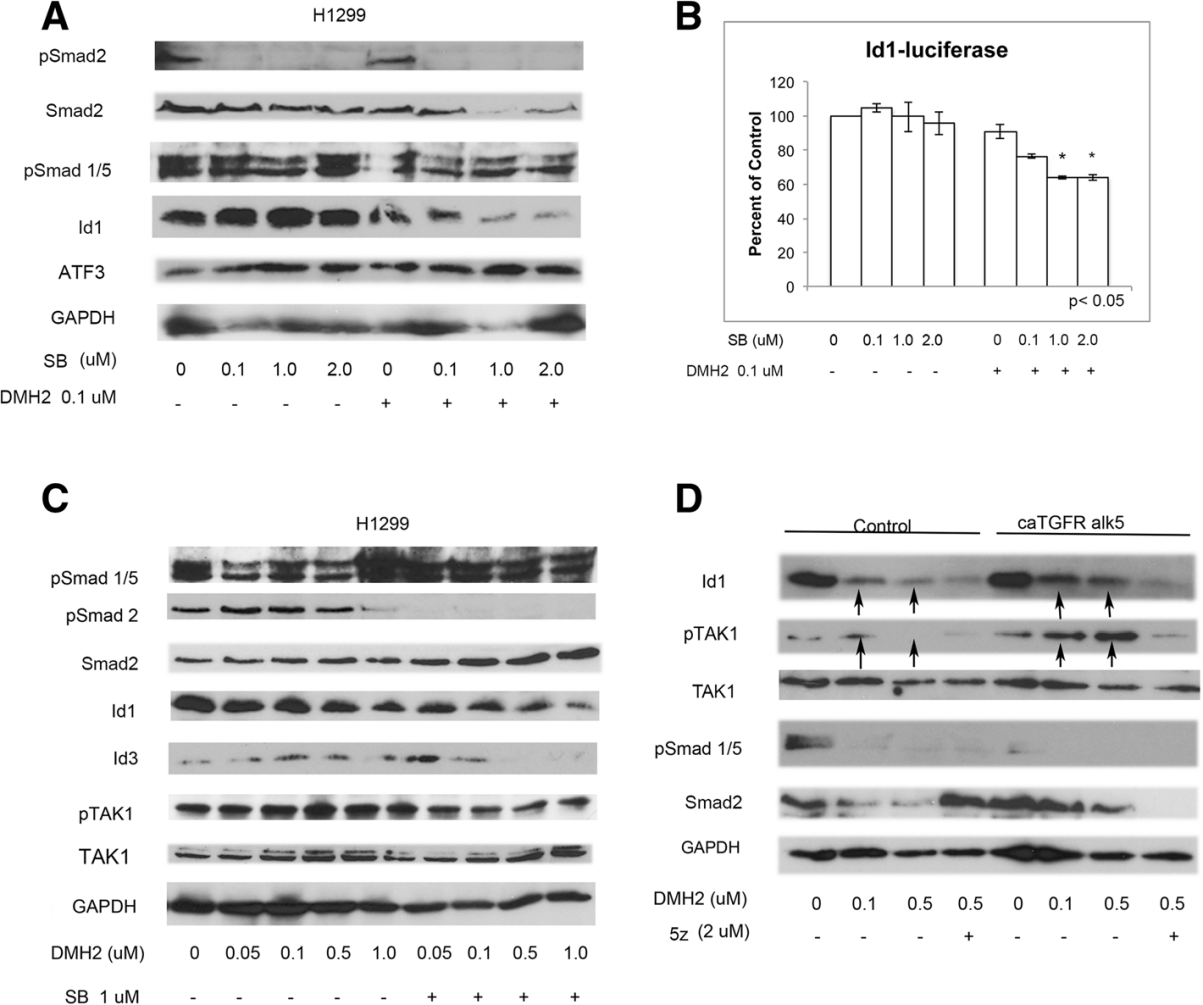
Inhibition of both BMP and TGFβ signaling enhances the downregulation of pTAK1 and Id1. **a** Western blot analysis of H1299 cells treated with increasing doses of the TGFβ inhibitor SB-505124 (SB) with and without DMH2. SB and DMH2 together enhanced the downregulation of Id1 (*n* = 3). (**b**) Id1-luciferase assay demonstrating decreased Id1 promoter activity only in cells treated with both SB and DMH2 (*n* = 2). (**c**) Western blot analysis of H1299 cells treated with 1 μM SB and increasing doses of DMH2 (*n* = 4). The combination of SB and DMH2 enhanced the downregulation of Id1, Id3, and pTAK1. (**d**) H1299 cells were transfected with constitutively active alk5 (ca alk5) or empty vector and treated with DMSO or DMH2 for 48 h (*n* = 3). Cells were also treated with or without 2 μM 5z for 24 h. Western blot shows that when BMP signaling is inhibited caAlk5 increases Id1 and pTAK1 expression that is attenuated with 5z. Arrows show increased expression of Id1 and pTAK1 in cells treated with caAlk5 and DMH2 compared to controls

We next examined TGFβ regulation of TAK1 and Id1 using the highly specific TGFβ antagonist SB-505124 (SB) [37] alone and in H1299 cells in which BMP signaling was suppressed with low dose of DMH2. SB alone caused a dose-related increase in the expression of Id1, suggesting TGFβ suppressed Id1 expression in H1299 cells (Fig. 4a). When BMP signaling was suppressed, SB had the opposite effect, now causing a dose-related decrease in the expression of Id1 (Fig. 4a). TGFβ signaling is reported to increase the expression ATF3, which is a co-repressor required for Smad3 to suppress the Id1 promoter [21]. In the absence of ATF3, Smad3 can activate the transcription of Id1. There were no significant change in the expression of ATF3 following suppression of BMP and TGFβ signaling, suggesting that ATF3 was not involved in the change of Id1 expression (Fig. 4a). SB alone caused an increase in the Id1 promoter activity in H1299 cells (Fig. 4b). When BMP signaling was suppressed with a low dose of DMH2, SB again had the opposite effect, now causing a dose-related decrease in Id1 promoter activity (Fig. 4b). We next asked whether inhibiting the feed-back activation of TGFβ signaling would enhance DMH2 downregulation of Id proteins and pTAK1. DMH2 caused a greater downregulation of the expression of Id1 and Id3 when used in combination with SB compared to DMH2 alone (Fig. 4c). In addition, there was a greater decrease in the expression of pTAK1 when DMH2 and SB were used in combination (Fig. 4c).

To further examine the role of TGFβ signaling causing a feedback activation of the BMP-TAK1-Id1 signaling cascade, H1299 cells were transiently transfected with vector control or constitutively active Alk5 (caAlk5) and treated with 0.1 and 0.5 μM of DMH2 for 48 h. Low doses of DMH2 were used so as not to inhibit TGFβ signaling. When BMP signaling was inhibited by DMH2, caAlk5 enhanced the activation of TAK1 and Id1 expression compared to cells treated with DMH2 alone (Fig. 4d). The increase in expression of Id1 and pTAK1 induced by caAlk5 together with 0.5 μM of DMH2 was antagonized by a 24-h treatment with 5Z (Fig. 4d). These studies support our observation that when BMP signaling is attenuated, activation of TGFβ signaling increases Id1 expression by activating TAK1.

### Antagonizing both BMP and TGFβ signaling enhances growth suppression

If inhibiting both BMP and TGFβ signaling enhances the downregulation of Id1, we would expect to have greater growth suppression when both were used together. We examined whether suppressing both BMP and TGFβ signaling enhanced growth suppression by treating cells with a BMP receptor inhibitor alone or in combination with SB. DMH2 alone caused significant growth suppression of the H1299 (Additional file 6: Figure S6A). The combination of DMH2 and SB caused a significantly greater decrease in cell number compared to either drug alone (Additional file 6: Figure S6A). SB alone had little effect on the growth of the H1299 cells (Additional file 6: Figure S6A). In the A549 cells, both DMH2 and SB-505124 alone caused growth suppression (Additional file 6: Figure S6B). A549 cells treated with both 2.5 μM DMH2 and 2.0 μM SB had significantly fewer cells than either compound alone (Additional file 6: Figure S6B). 2.5 μM of DMH1 combined with 1.0 μM or 2.0 μM SB-505124 caused significantly greater growth suppression than either compound alone (Additional file 6: Figure S6D).

### DMH2 induction of cell death involves the downregulation of pTAK1

**Fig. 5:**
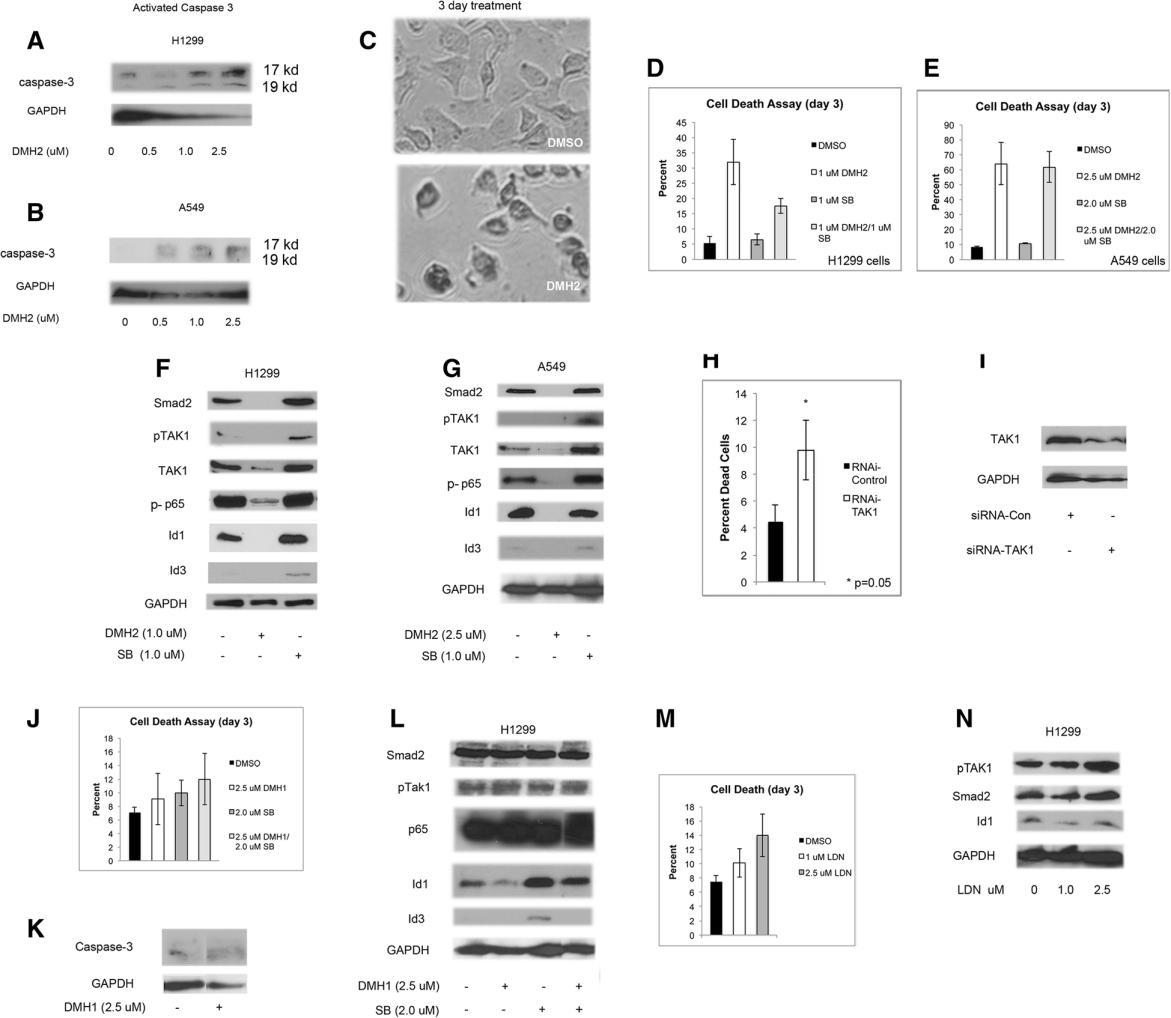
DMH2 induction of cell death involves the downregulation of pTAK1. **a**, **b** Western blot of H1299 and A549 cells treated with DMH2 for 48 h demonstrating an increase in activated caspase-3. (**c**) Representative images of H1299 cells treated with 1 μ DMH2 for 3 days demonstrating significant cell shrinkage and chromatin condensation. (**d**, **e**) H1299 and A549 cells were treated with DMH2 alone or in combination with SB for 3 days and cell death was determined. (**f**, **g**) Western blot analysis of cells treated with DMH2 or SB for 3 days. (**h**) Knockdown of TAK1 was performed in H1299 cells using siRNA. The percentage of dead cells was determined after 3 days. (**i**) Western blot analysis showing knockdown of TAK1 in H1299 cells. (**j**, **k**) DMH1 alone or in combination with SB did not cause significant cell death in H1299 cells after 3 days (**k**) and did not induce the activation of caspase-3. (**l**) Western blot analysis demonstrating DMH1 alone or in combination with SB did not show significant regulation of Smad2, pTAK1, or p65. (**m**) LDN induced little cell death compared to DMH2 and (**n**) did not downregulate Smad2 or TAK1 by Western blot analysis. All experiments were performed at least 3 times

We next examined if the regulation of TAK1 was a mechanism by which BMP inhibitors induced death of lung cancer cells. DMH2 induced expression of activated caspase-3 at 48 h (Fig. 5a and b) and by 72 h cells showed significant shrinkage and chromatin condensation (Fig. 5c). These studies support our observation that DMH2 induces apoptotic cell death. DMH2 induced significant cells death of H1299 and A549 cells after 3 days (Fig. 5d and e). The addition of SB together with DMH2 did not cause further cell death (Fig. 5d and e). Examining the mechanisms by which DMH2 induces cell death, we found that DMH2 caused a very significant decrease in the expression of Smad2 in both H1299 and A549 cells (Fig. 5f and g). This was associated with a decrease in expression of TAK1 and the phosphorylation of its downstream target the NF-kappa B subunit p65 [28] (Fig. 5f and g). Knockdown of TAK1 with siRNA in the H1299 cells caused cell death suggesting its downregulation is a mechanism by which DMH2 induces cell death (Fig. 5h and i). DMH1 (2.5 μM) alone or when combined with SB caused little cell death in comparison to DMH2 (Fig. 5j). DMH1 alone or in combination with SB also did not activate caspase-3 or downregulate Smad2, pTAK1, or p-p65 after 3 days (Fig. 5k and l). LDN is an inhibitor of both the BMP and TGFβ receptors. LDN also caused little cell death and downregulation of Smad2 and TAK1 in comparison to DMH2 after 3 days (Fig. 5m and n). These studies supports that DMH2 downregulation of TAK1 is involved in induction of cell death but that additional mechanism(s) may also be involved in addition to inhibition of BMP type I receptors and TGFβ signaling.

### DMH2 induction of cell death involves XIAP, which regulates the activity of TAK1

**Fig. 6:**
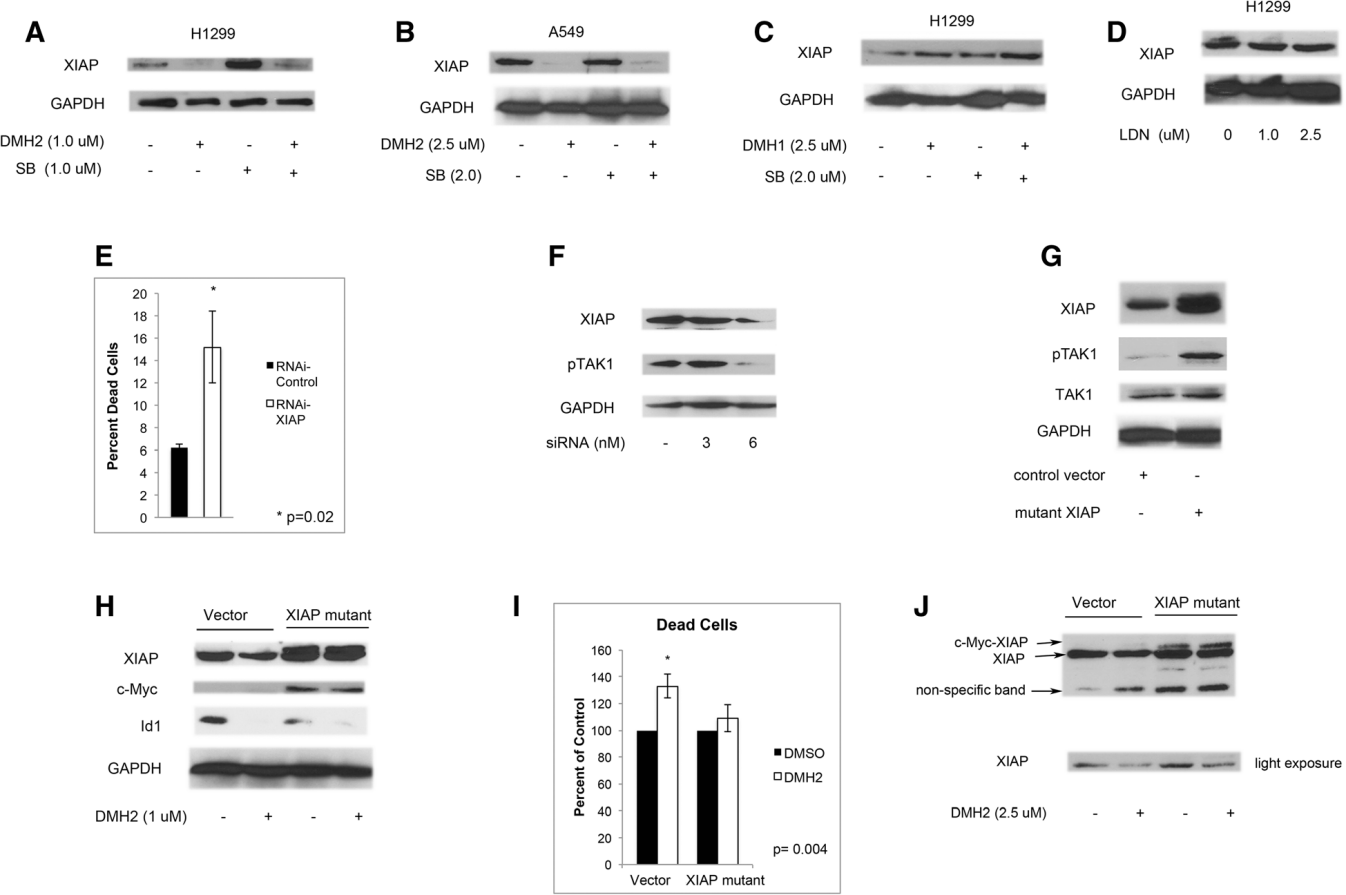
DMH2 regulation of cell death involves the downregulation of XIAP, which regulates the activity of TAK1. **a**-**d** Western blot analysis of cells treated with DMH2, DMH1, or LDN and in combination with SB for 3 days. Only DMH2 induced the downregulation of XIAP. (**e**) The knockdown of XIAP was performed using siRNA in H1299 cells and after 3 days percentage of dead cells determined. (**f**) Western blot analysis showing that knockdown of XIAP decreases the expression of pTAK1. (**g**) H1299 cells were transiently transfected with mutant XIAP with ubiquitination sites removed. Western blot analysis after 48 h demonstrates increased expression of pTAK1. (**h**) H1299 cells were transiently transfected with control vector and mutant XIAP containing a N-terminal c-Myc epitope. Cells immediately after transfection were treated with DMSO or DMH2 for 3 days. Western blot demonstrating that DMH2 decreased expression of wild type XIAP but not of mutant XIAP. (**i**) H1299 cells were stably transfected with vector control and mutant XIAP. Cells were treated with and without DMH2 for 3 days and the percentage of cell death was determined. DMH2 only induced cell death in the vector control cells. All experiments were performed at least 3 times. (**j**) Western blot analysis of stable transfected cells treated with DMSO or DMH2 for 3 days. The bottom panel shows a shorter exposure of the panel above (*n* = 2)

During embryonic development the BMP signaling cascade mediates the activation of TAK1 by stabilizing the expression of XIAP through Smad1/5 independent mechanism [38]. DMH2 caused a significant decrease in the expression of XIAP in both the H1299 and A549 cells after 3 days (Fig. 6a and b). LDN and DMH1 alone or with SB-505124 did not downregulate the expression of XIAP after 3 days (Fig. 6c and d). Knockdown of XIAP with siRNA in the H1299 cells induced cell death (Fig. 6e) and caused a decrease in expression of pTAK1 (Fig. 6f). Since BMP type I and type II regulate the ubiquitination of XIAP [26], mutant XIAP that has had its lysine ubiquitination sites mutated [39] was transiently transfected into the H1299 cells. H1299 cells expressing mutant XIAP had a higher expression of pTAK1 compared to vector control cells (Fig. 6g). DMH2 decreased the expression of Id1 and wild type XIAP but not the mutant XIAP (Fig. 6h). Mutant XIAP and vector control cDNA were stably transfected into the H1299 cells. H1299 cells expressing mutant XIAP were resistant to cell death induced by DMH2 (Fig. 6i). In cells stably expressing mutant XIAP, DMH2 also decreased the expression of wild type XIAP but not mutant XIAP (Fig. 6j). These studies show that XIAP is an upstream regulator of TAK1 in lung cancer cells and that the downregulation of XIAP is required for DMH2 induced cell death.

### Inhibition of BMP type I receptors and TGFβ signaling induce cell death when XIAP is downregulated

**Fig. 7:**
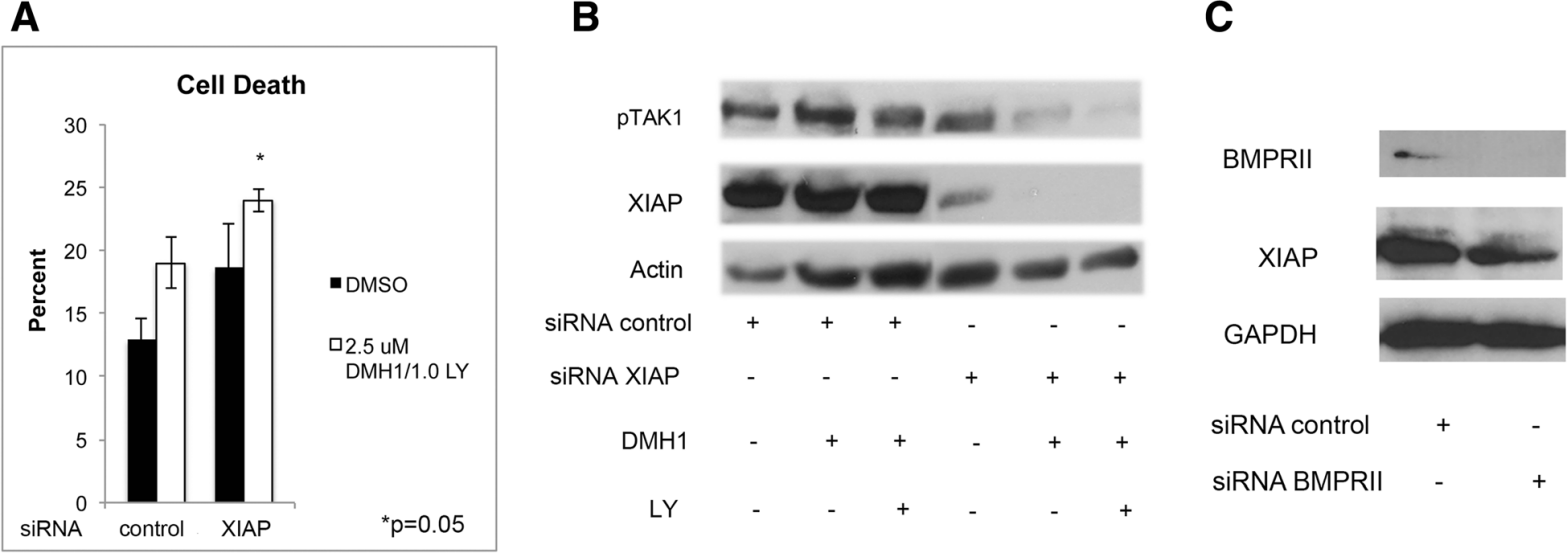
DMH1 together with TGFβ inhibition induces cell death and downregulates pTAK1 that is greatest when XIAP is knocked down. BMPRII regulates XIAP expression. **a** H1299 cells were treated with control siRNA and siRNA targeting XIAP and treated with DMH1 and the TGFβ inhibitor LY2109761 (LY) (*n* = 3). Cells with knockdown of XIAP and treated with DMH1 and LY demonstrated the greatest percentage of cell death. (**b**) Western blot analysis showing that H1299 cells with knockdown of XIAP and treated with both DMH1 and LY had the greatest decrease in expression of pTAK1 (*n* = 4). (**c**) Western blot analysis demonstrating knockdown of BMPRII in H1299 cells with siRNA decreases the expression of XIAP (*n* = 4)

To further examine the role of the TGFβ signaling cascade in regulating cell death, XIAP was knocked-down in H1299 cells then cells were treated with the inhibitor of the BMP type I receptors (DMH1) and with the TGFβ antagonist LY2109761 (LY) [40]. The greatest cell death and decrease in pTAK1 expression occurred in XIAP knockdown cells treated with a combination of DMH1 and LY (Fig. 7a and b). These data further support that the downregulation of XIAP and inhibition of TGFβ signaling are mechanisms by which DMH2 inhibits TAK1 and induces cell death.

#### BMPRII regulates XIAP expression

Since DMH2 inhibits BMPRII (Table 1), we examined whether the downregulation of BMPRII effects the expression of XIAP in lung cancer cells. The knockdown of BMPRII by siRNA decreased the expression of XIAP in the H1299 cells (Fig. 7c). These data suggest that DMH2 inhibition of BMPRII is an important mechanism by which it decreases the expression of XIAP and induces cell death in cancer cells. Scheme 1 demonstrates the interactive pathways mediated by the BMP and TGFβ signaling pathways to regulate survival of cancer cells.

## Discussion

The BMP signaling pathway is a potent direct regulator of the transcription of Id1, Id2, and Id3 during development as well as in cancer cells [11–16]. The decrease in the expression of Id proteins is an importance mechanism by which BMP inhibitors regulate anti-tumor effects [16]. Numerous studies have demonstrated the importance of Id proteins in cancer by regulating invasion, metastasis, angiogenesis, cell growth and survival [4, 14, 41–43]. Surprisingly, our xenograft studies showed that DMH2 caused an increase in the expression of Id1. The increase in the expression of the Id1-luciferase reporter was consistent with a feedback loop activating the Id1 promoter. The poor pharmacokinetics of DMH2 with high plasma protein binding and rapid clearance suggest that DMH2 would have provided only weak inhibition in tumor xenografts. Our studies suggested that with low levels of inhibition of BMP signaling, feedback loops activated the transcription of Id1. Our studies support that the feedback activation of Id1 upon inhibition of BMP signaling did not occur through the Src or MEK-1/2 signaling pathways.

We found that the inhibition of BMP signaling in lung cancer cells causes an increase in the expression of activated TAK1 and TGFβ. TAK1 has been shown to cause a feedforward activation of BMP signaling in progenitor cells by binding to Smad-1/5 [27]. We show that TAK1 also activates pSmad-1/5 and increases the expression of Id1 in lung cancer cells. Consistent to reported literature in benign cells, both the BMP and TGFβ signaling cascades activate TAK1 in lung cancer cells. Importantly, our studies suggest that the inhibition of both BMP and TGFβ pathways will enhance the downregulation of TAK1 and Id1 expression as well as having a greater anti-tumorigenic response. TGFβ signaling predominantly inhibits the transcription of Id1 through its activation of Smad2 and Smad3. Smad3, but not Smad2, has been shown to cause a transient increase in the transcription of Id1 [22]. Smad3 inhibition of the Id1 promoter requires co-binding with ATF3 [21]. In the absence of ATF3, Smad3 activates the transcription of Id1 [21]. The increased expression of Id1 in our studies does not appear to be from TGFβ signaling changing from a negative to a positive transcriptional regulator of Id1 since there was no significant change in the expression of ATF3 following the inhibition of TGFβ or BMP signaling.

Our studies also show that in addition to inhibiting TGFβ signaling, DMH2 potency was dependent on the downregulation of XIAP. XIAP is an upstream regulator of TAK1 during development. We found that XIAP was also an upstream regulator of TAK1 in lung cancer cells. The TGFβ signaling pathway has been shown to activate TAK1 independently of XIAP. TGFβ receptors bind to necrosis factor receptor-associated factor 4 (TRAF4) [44] and TRAF6 [45] leading to the activation of TAK1. These studies support inhibiting both the TGFβ and BMP signaling pathways to inhibit TAK1 and its downstream survival pathway. Examining differences in IC50 for the BMP and TGFβ receptors, we found only DMH2 caused inhibition of BMPRII. Developmentally, both the type I and type II BMP receptors have been shown to stabilize the expression of XIAP [25, 26]. Our studies suggest that inhibition of BMPRII is required to cause significant downregulation of XIAP. TGFβ signaling has also been shown to increase the expression of XIAP [46, 47]. Although not specifically addressed in our studies, the inhibition the BMP type I and/or TGFβ signaling may also have contributed to DMH2 induced downregulation of XIAP.

XIAP is the most potent inhibitor of apoptosis [29]. XIAP binds and inactivates effector caspases-3 and 7 and initiator caspase-9 [48]. XIAP also functions as an E3 ligase, inducing the degradation of caspases via the proteasome system [48]. TAK1 is also a potent inhibitor of cell death [28], which is mediated through its activation of NF-κB [28] and by preventing reactive oxygen species production [49]. NF-κB inhibits cell death by inducing the expression of cellular FLICE-like protein (c-FLIP) and cIAPs [28]. Smac mimetic Inhibitors of XIAP, c-IAP-1, and c-IAP-2 can induce significant cell death in some cancer cell lines. Resistance to Smac mimetics has been shown to occur through a NF-κB mediated upregulation of c-FLIP [50]. Knockdown of c-FLIP together with XIAP was sufficient to induce significant cell death in resistant cancer cell lines [50]. These studies demonstrate the importance of inhibiting NF-κB or its downstream pathways to induce cell death following inhibition of XIAP. Id1 has also been shown to promote survival of cancer cells by activating NF-kappa B [51]. Overexpression of Id proteins decreased DMH2 induced cell death of H1299 cells [16]. These studies suggest that suppression of XIAP, TAK1, and Id proteins may be required for a BMP inhibitor like DMH2 to induce significant death of cancer cells.

## Conclusions

This study suggests a paradigm in which the BMP and TGFβ signaling pathways regulate the survival of cancer cells that involves both Smad dependent and independent signaling. We show that inhibition of the BMP signaling cascade with small molecule inhibitors decrease the expression of the potent inhibitors of cells death, XIAP and TAK1 through evolutionary conserved pathways. We provide evidence that a small molecule receptor inhibitor that targets both the BMP and TGFβ receptors enhances the downregulation of TAK1 by preventing the feedback activation of TAK1 by TGFβ signaling, which occurs following the suppression BMP signaling alone. Our studies suggest the that an inhibitor targeting BMP and TGFβ type I and type II receptors best inhibits anti-apoptotic pathways to induce death of lung cancer cells. The high level of expression of BMP proteins in cancer with little expression in normal tissue, and the ability of BMP inhibitors to regulate several anti-apoptotic pathways suggest that targeting the BMP signaling cascade may provide an effective strategy for the treatment of lung cancer.

## Methods

Mice studies were approved by the Rutgers, Robert Wood Johnson Medical School Institutional Animal Care and Use Committee.

### Plasmids

Constitutively active alk3 and alk6 constructs in mammalian vectors were gifts from Joan Massague (New York, New York). Constitutively active alk5 was a gift from Fang Liu (Rutgers-New Jersey Medical School). The Id1/luciferase promoter was a gift from Dr. Desprez (California Pacific Medical Center). Plasmids pc DNA3-XIAP-Myc and pcDNA3-XIAP-Myc K322/D28 were gifts from Guy Salvesen (Addgene plasmid # 11833 and plasmid # 11834).

### Cell culture and reagents

The A549 and H1229 lung cancer cell lines were cultured in Dulbecco’s Modified Eagle’s medium (DMEM, Sigma Aldrich, St Louis, MO, USA) with 5 % fetal bovine serum (FBS), 1 % antibiotic/antimycotic, and 1 % L-glutamine [52]. The lung cancer cell lines H157, H727, U1752, and H358, and H865 were cultured in 90 % RPMI and 10 % FCS. The cell lines were obtained from ATCC and from Malcolm Brock, Johns Hopkins University.

#### BMP inhibitors

Dorsomorphin (compound C) was purchased from Sigma Aldrich (St. Louis, MO) and DMH1, and LDN-193189 (LDN) were purchased from Selleckchem (Houston, TX.) DMH2 was synthesized at Rutgers- New Jersey Medical School (Dave Augeri). TGFβ inhibitors LY2109761 and SB-505124 were purchased from Selleckchem and Sigma Aldrich (St. Louis, MO) respectively. The Tak1 inhibitor 5Z-7-oxozeaenol was purchased from Sigma Aldrich.

### Transient gene knockdown and transfections

Select siRNA were used to target the type I BMP receptors alk2, alk3, and alk6 (Life Technologies). The ID numbers of the siRNA are: alk2 (s974), alk3 (s281), alk6 (s2042). The knockdown of the BMP receptors with these siRNAs have been previously validated on the H1299 cells and confirmed on the blots used in this study [16]. Validated siRNA XIAP (S1456), siRNA TAK1, and siRNA BMPRII were purchased from Life Technologies. Silencer Select Negative Control siRNA (4390843) was used to confirm specificity of each targeted knockdown.

Cells were transfected with siRNA using a Nucleofector II (Amaxa Biosystems, Gaitherburg, MD) using the manufacture’s Nucleofector kit T. Studies using cell death assay cells were transfected with Lipofectamine 3000. A total of 30nM of siRNA was used for alk2 and alk3 [16] and 20 nM for alk6 and 30nM for XIAP. Plasmid cDNA was transfected at 2.5ug.

### Western blot analysis

Total cellular protein was prepared using RIPA buffer containing a protease inhibitor cocktail and protein concentration was measured using the BCA assay as described [4]. In brief, protein was analyzed by SDS-PAGE, transferred to nitrocellulose (Schleicher and Schuell, Keene, NH). After blocking, the blots were incubated overnight at 4 °C with the appropriate primary antibody in Tris-buffered saline with 1 % Tween (TBST) and 5 % BSA. Secondary antibodies were applied for 1 h at room temperature. Specific proteins were detected using the enhanced chemiluminescence system (Amersham, Arlington Heights, IL). The primary antibodies that were used were rabbit monoclonal anti-pSmad 1/5, rabbit monoclonal anti-pSmad2, rabbit monoclonal anti-Smad2, rabbit monoclonal anti-pTAK1, rabbit monoclonal XIAP, rabbit anti-monoclonal p-p65, rabbit monoclonal anti-activated caspase-3, rabbit polyclonal anti-BMPRII, and rabbit monoclonal anti-EGR-1 (Cell signaling Technology, Danvers MA), Rabbit monoclonal anti-TAK1 (Invitrogen, Grand Island NY), rabbit anti-actin, an affinity isolated antigen specific antibody (Sigma, Saint Louis, MO), rabbit monoclonal anti-Id1 and rabbit monoclonal anti-Id3 (Calbioreagents, San Mateo, CA), rabbit polyclonal anti-GAPDH (Sigma, St. Louis, MO) and rabbit polyclonal pMEK-1/2 (Cell Signaling, Danvers MA).

### Luciferase assays

H1299 cells were stably transfected with the Id1-promoter luciferase reporter. Treated cells were lysed with 1X luciferase lysis buffer (Promega). Samples were added to luciferase assay substrate (Promega) and luminsescence measured by the TD-20/20 Luminometer (Turner Designs/Turner BioSystems, Sunnyvale, CA). Control samples included luciferase assay substrate alone and luciferase assay substrate plus 1X reporter lysis buffer.

### Tumor xenografts

H1299 cells were stably transfected with the Id1 promoter-luciferase reporter. Five million cells were injected subcutaneously into the flank of NCI female nude mice. After approximately 12 days the mice were anesthetized and the luciferase activity measured. Baseline luciferase activity was obtained just prior to the injection of inhibitors. Mice were injected IP with 150 mg/kg luciferin-D and after a 15-min uptake period tumors were imaged with IVIS Spectrum and analyzed using Living Image software. Mice were injected IP with DMSO, 3 mg/kg DMH2, or 3 mg/kg LDN and after 4 h luciferase activity determined. Two days later, baseline luciferase activity again determined and mice were injected IP with DMSO, 3 mg/kg DMH2, or 3 mg/kg LDN every 8 h for three doses and luciferase activity was measured 4 h after the last dose. In separate animal experiment, mice with established tumors were treated with DMSO or 3 mg/kg DMH2 twice daily for 48 h and luciferase activity compared to baseline. The tumors were harvested on ice, homogenized, and placed in lysis buffer. In a separate experiment, mice with established tumor xenografts were injected IP with DMSO, 3 mg/kg DMH2, or 9 mg/kg DMH2 every 12 h for 9 days and tumors were then harvested.

### Cell counts

Cells were plated into 6 well plates at 10^5^ cells per well and treated with 1 μM DMSO or antagonist for 7 days. The cells were detached with trypsin, stained with trypan blue, and the number of live cells counted using a hemocytometer.

### Cell death assay

Cells were plated in 6 well plates with 10^6^ cells per well. Three days following treatment with inhibitor(s) and/or transfection the adherent and floating cells were harvested and incubated with 0.1 mg/ml of ethidium bromide. Cells that die by necrosis or apoptosis lose membrane integrity and take up ethidium bromide [53]. Immediately after staining approximately 100 cells were counted and the percentage of cells that took up ethidium bromide was determined.

### In vitro kinase IC_50_

The IC_50_ of DMH2 for alk2, alk3, alk6, alk5, BMPRII, and TGFβ were performed at Reaction Biology Corporation (Malvern, PA). This was a 10-point assay starting from 100 μM to 100 nM performed in duplicate. The ATP concentration was 10 Micromolar.

### Plasma protein binding

Human and mouse protein plasma binding of DMH2 was performed using equilibrium dialysis (Sai Life Sciences Limited, Pune India).

### Pharmacokinetics

The pharmacokinetics of DMH2 was examined in maie BALB/c mice following intravenous and oral administration (Sai Life Sciences Limited, Pune India). Three mice at each time point were dosed with a 2 mg/kg tail vein injection and 10 mg/kg p.o. Blood samples were taken 0.08, 0.25, 0.5, 1, 2, 4, 8, 12, and 24 h (i.v.) and 0.25, 0.5, 1, 2, 4, 6, 8, 12, and 24 h (p.o.). Plasma half-life, clearance, and volume of distribution were then determined.

### Statistical analysis

The mean of the control group was compared to the mean of each treated group using a paired student *t*-test assuming unequal variances. Differences with *p* values <0 .05 were considered statistically significant.

## Additional files

Additional file 1: Figure S1. DMH2 decreases Id1 expression and growth of lung cancer cell lines in vitro. (A) Western Blot analysis of panel of cell lines in cell culture treated with 1 μM DMH2 for 48 h demonstrating a downregulation of Id1. (B) Cell counts of cell lines treated with 1 μM DMH2 for 7 days. Data is depicted as percent of vehicle control. Experiments were performed 3 times. (TIF 749 kb) Additional file 2: Figure S2. Low doses of DMH2 increases Id1 expression in A549 cells. Western blot analysis of A549 cells in cell culture treated with increasing doses of DMH2 for (A) 24 and (B) 48 h. Non-specific band from the same Western blot was used as a loading control. Experiments performed at least 3 times. (TIF 2680 kb) Additional file 3: Figure S3. Pharmacokinetics of DMH2. (A) Determination of DMH2 plasma concentration following IV and PO injections demonstrates rapid clearance. (B) The unbound free fraction of DMH2 was calculated from plasma concentration over time from IV injection in mice assuming 98.3 % was bound to plasma proteins. (TIF 1187 kb) Additional file 4: Figure S4. MEK-1/2 and Src signaling do not cause feedback activation of Id1 following inhibition of BMP signaling. (A-B) Western blot of tumor xenografts treated with BMP inhibitors for 24 h and 9 days. (C) Western blot analysis of H1299 cells treated with DMH2 for 24 and 48 h. (D) Western blot analysis of A549 cells treated with DMH2 for 48 h. (E) H1299 Id1-luc cells were treated with DMH2 or PD0325901 (PD) alone or in combination for 48 h and luciferase activity determined. (F-G) H1299 and A549 cells were treated with DMH2 or PD alone, or in combination and the number of live cells determined after 7 days. (E-G) Data depict the mean as the percent of control. Experiments were performed at least 3 times. (TIF 9413 kb) Additional file 5: Figure S5. DMH2 is more potent than DMH1. H1299 Id-1 luc cells were treated with increasing concentrations of DMH1 or DMH2 for 48 h and luciferase activity was determined. The data represents the mean of at least 4 experiments. (TIF 359 kb) Additional file 6: Figure S6. Inhibition of both BMP and TGFβ signaling enhances growth suppression (A–D). Cell lines were treated with DMH2 or DMH1 alone and with SB for 7 days and cell counts were performed. The studies represent the mean of at least 3 independent experiments. P values were determined comparing cells treated with DMH2 and SB alone to cells treated with both inhibitors. (TIF 2411 kb)

## Abbreviations

5Z: 7-oxozeaenol (5Z)
AMP-kinase: adenosine monophosphate-activated protein kinase
BMP: bone morphogenetic protein
Egr-1: early growth response protein
Id1: Inhibitor of differentiation
LDN: LDN-193189
LY: LY2109761
MEK-1/2: mitogen-activated protein kinases
NSCLC: non-small cell lung
SB: SB-505124
siRNA: short interfering RNA
TAB: TAK1 binding protien
TAK1: TGFβ activated kinase
TGFβ: Transforming Growth Factor Beta
TRAF4: necrosis factor receptor-associated factor 4
TRAF6: necrosis factor receptor-associated factor 6
VEGF II: vascular endothelial growth factor
XIAP: X-link inhibitor of apoptosis protein
