# Supplementary figures and images for: Inhibition of BMP and of TGFβ receptors downregulates expression of XIAP and TAK1 leading to lung cancer cell death
Source: Mol Cancer. 2016 Apr 6;15:27. doi: 10.1186/s12943-016-0511-9 (PMC4822253; doi:10.1186/s12943-016-0511-9)

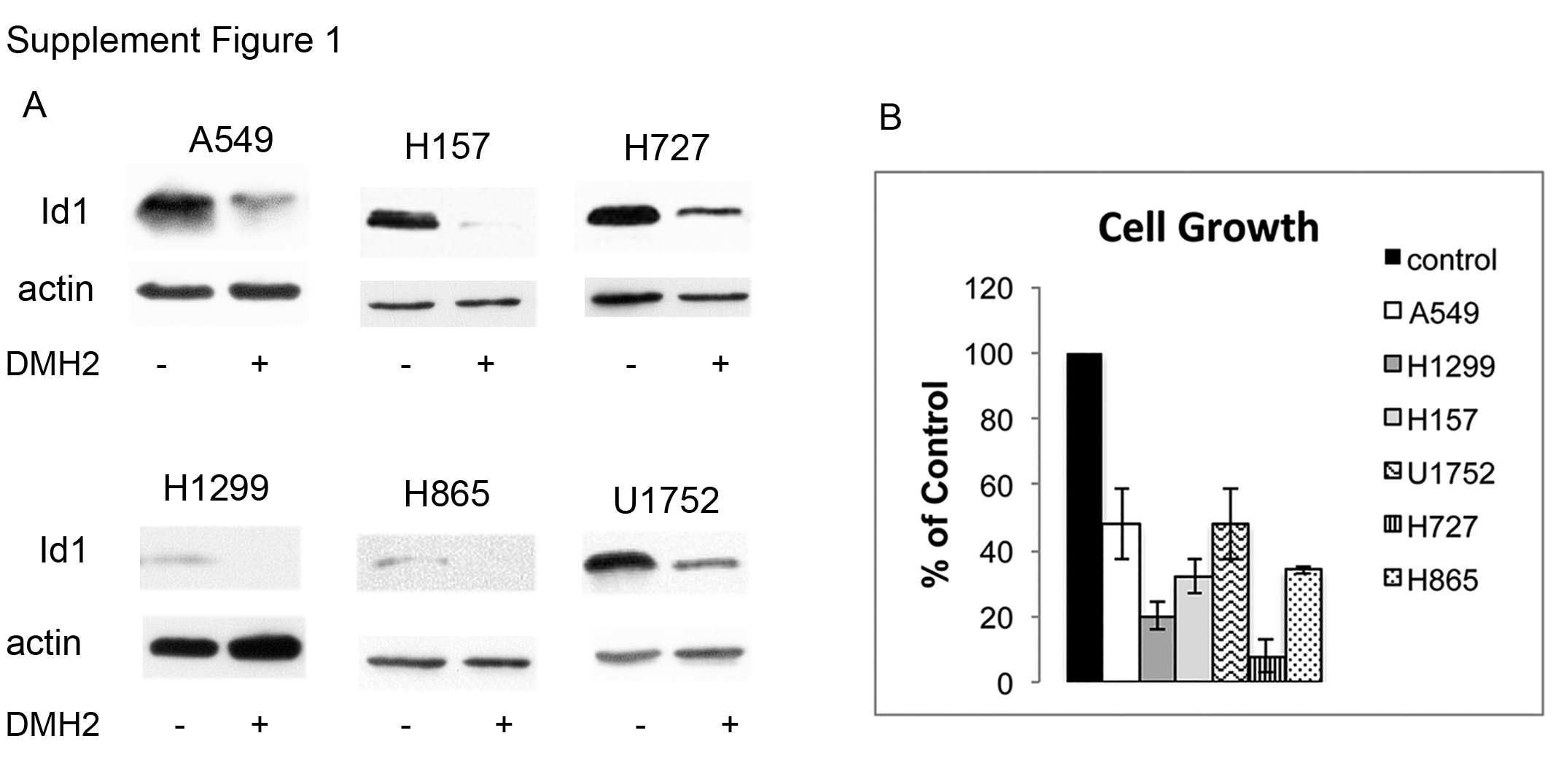

Supplement: Additional file 1: Figure S1. — DMH2 decreases Id1 expression and growth of lung cancer cell lines in vitro. (A) Western Blot analysis of panel of cell lines in cell culture treated with 1 μM DMH2 for 48 h demonstrating a downregulation of Id1. (B) Cell counts of cell lines treated with 1 μM DMH2 for 7 days. Data is depicted as percent of vehicle control. Experiments were performed 3 times. (TIF 749 kb) [file 12943_2016_511_MOESM1_ESM.tif]

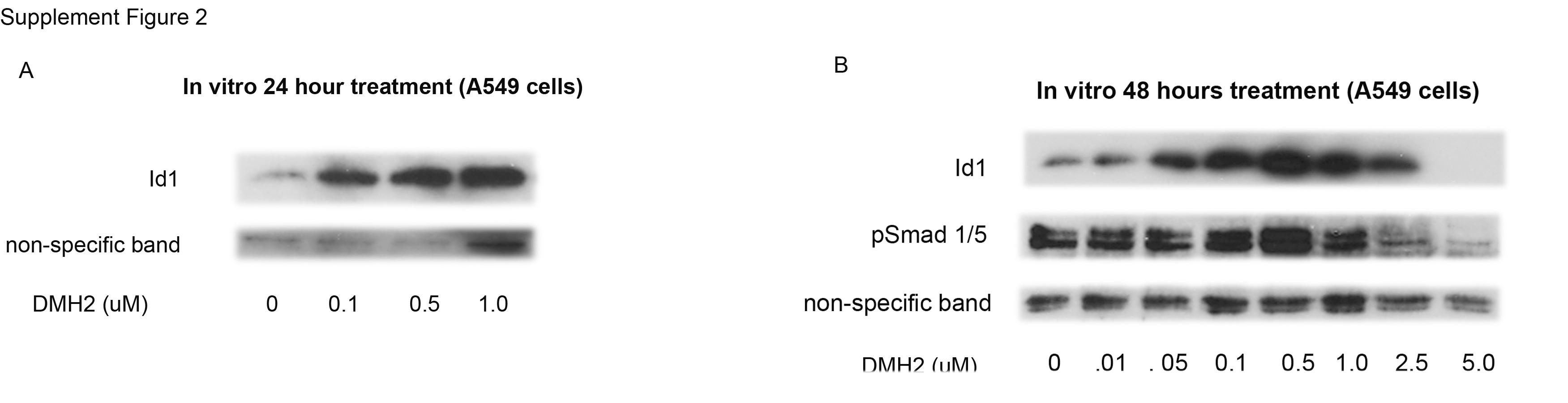

Supplement: Additional file 2: Figure S2. — Low doses of DMH2 increases Id1 expression in A549 cells. Western blot analysis of A549 cells in cell culture treated with increasing doses of DMH2 for (A) 24 and (B) 48 h. Non-specific band from the same Western blot was used as a loading control. Experiments performed at least 3 times. (TIF 2680 kb) [file 12943_2016_511_MOESM2_ESM.tif]

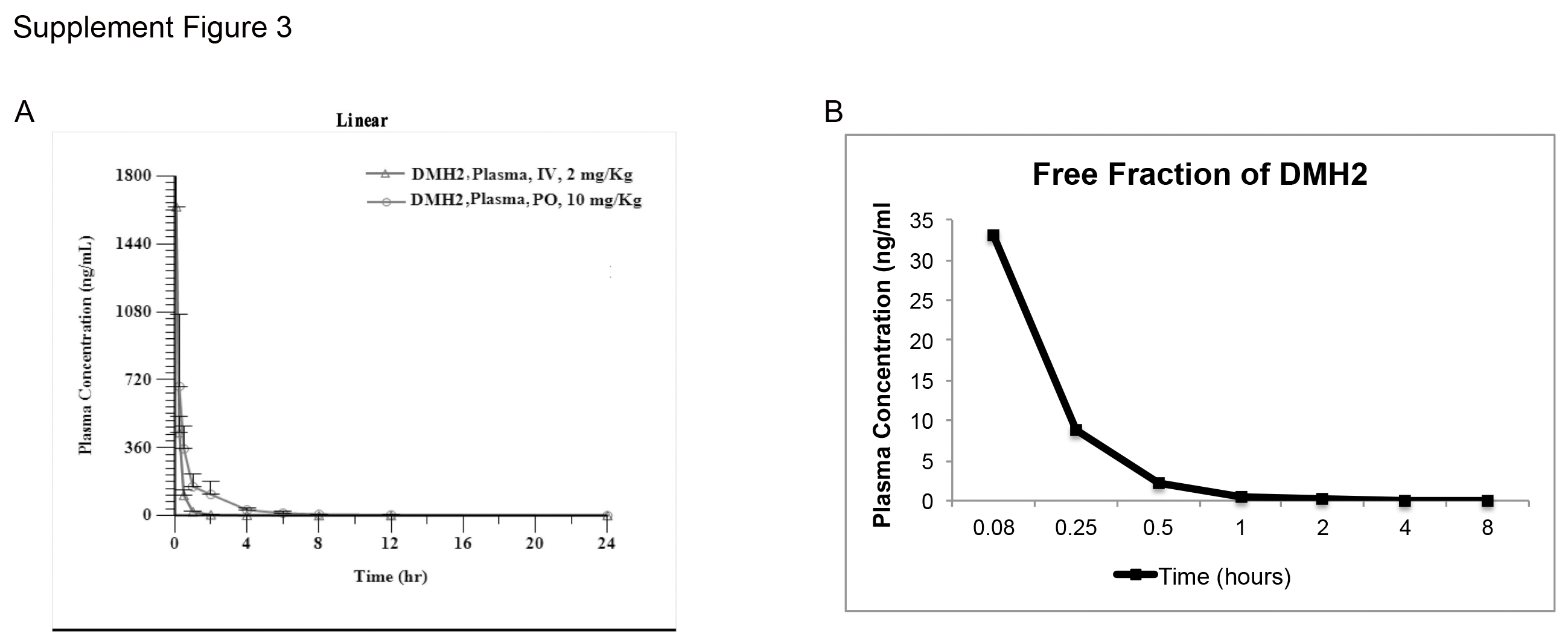

Supplement: Additional file 3: Figure S3. — Pharmacokinetics of DMH2. (A) Determination of DMH2 plasma concentration following IV and PO injections demonstrates rapid clearance. (B) The unbound free fraction of DMH2 was calculated from plasma concentration over time from IV injection in mice assuming 98.3 % was bound to plasma proteins. (TIF 1187 kb) [file 12943_2016_511_MOESM3_ESM.tif]

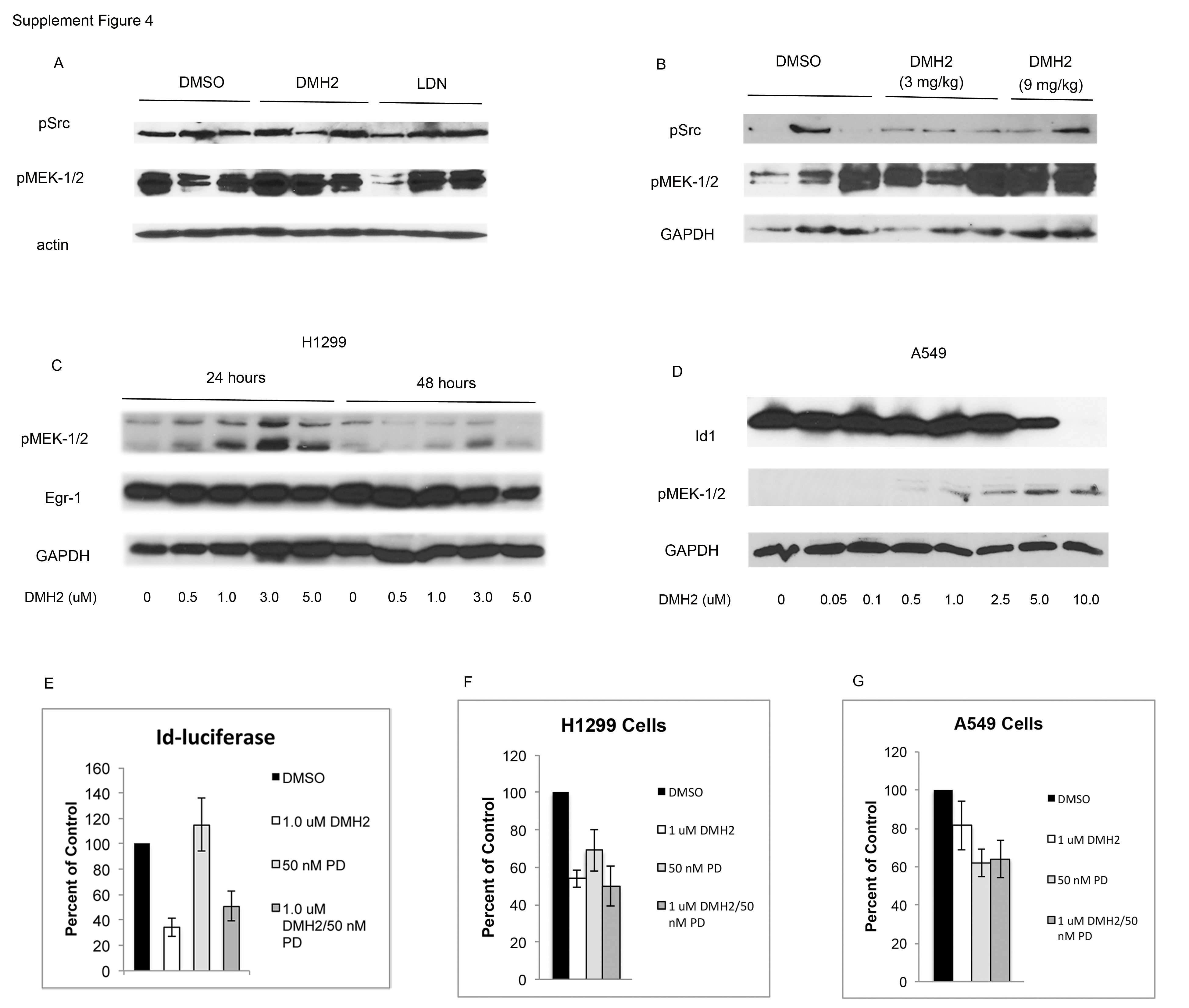

Supplement: Additional file 4: Figure S4. — MEK-1/2 and Src signaling do not cause feedback activation of Id1 following inhibition of BMP signaling. (A-B) Western blot of tumor xenografts treated with BMP inhibitors for 24 h and 9 days. (C) Western blot analysis of H1299 cells treated with DMH2 for 24 and 48 h. (D) Western blot analysis of A549 cells treated with DMH2 for 48 h. (E) H1299 Id1-luc cells were treated with DMH2 or PD0325901 (PD) alone or in combination for 48 h and luciferase activity determined. (F-G) H1299 and A549 cells were treated with DMH2 or PD alone, or in combination and the number of live cells determined after 7 days. (E-G) Data depict the mean as the percent of control. Experiments were performed at least 3 times. (TIF 9413 kb) [file 12943_2016_511_MOESM4_ESM.tif]

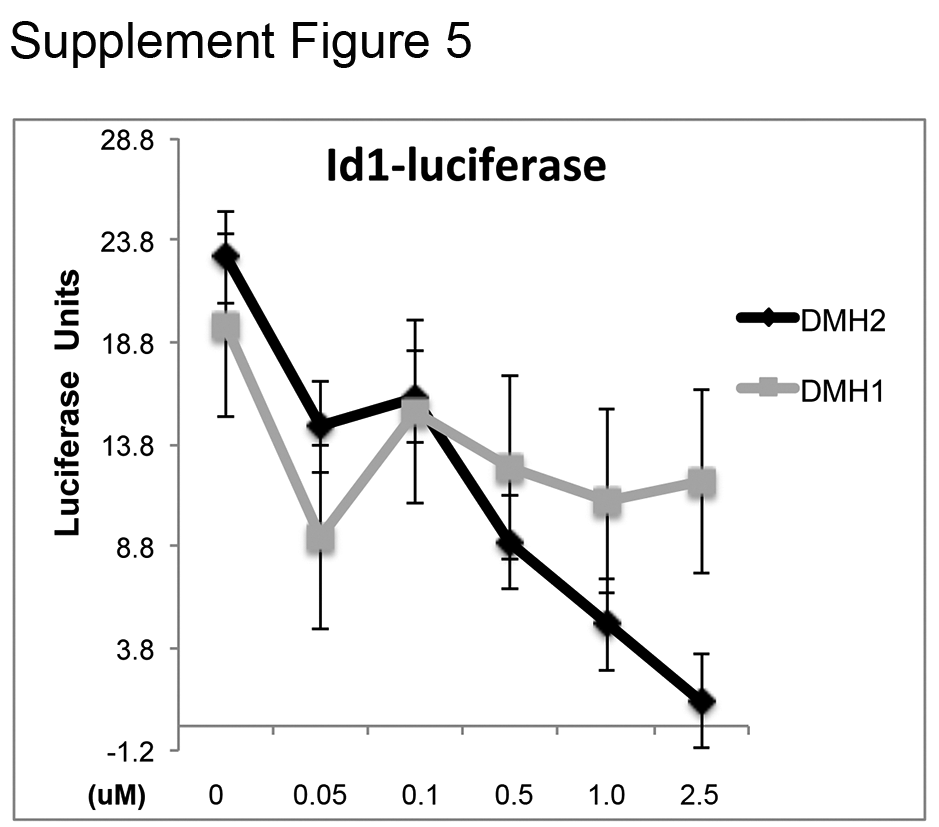

Supplement: Additional file 5: Figure S5. — DMH2 is more potent than DMH1. H1299 Id-1 luc cells were treated with increasing concentrations of DMH1 or DMH2 for 48 h and luciferase activity was determined. The data represents the mean of at least 4 experiments. (TIF 359 kb) [file 12943_2016_511_MOESM5_ESM.tif]

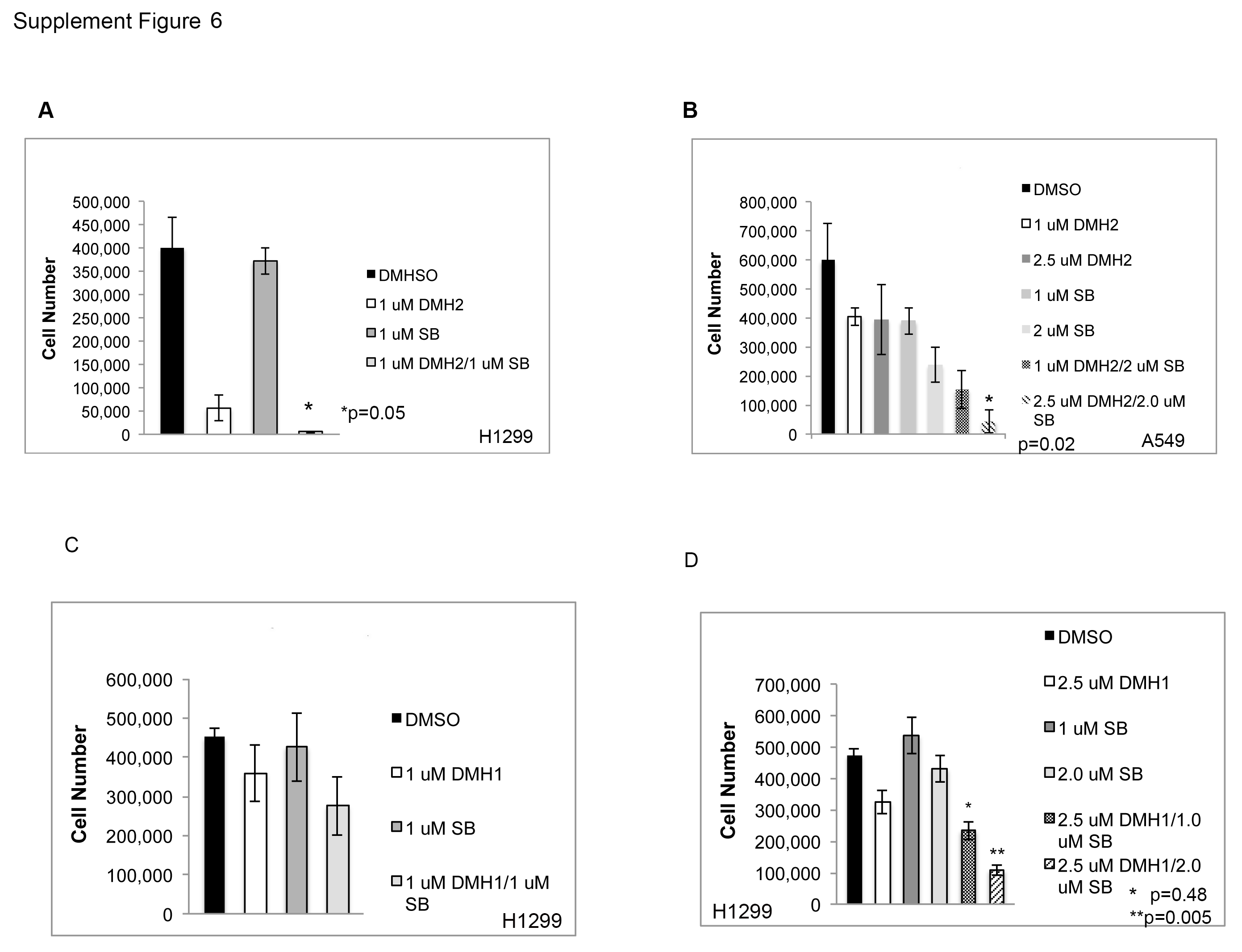

Supplement: Additional file 6: Figure S6. — Inhibition of both BMP and TGFβ signaling enhances growth suppression (A–D). Cell lines were treated with DMH2 or DMH1 alone and with SB for 7 days and cell counts were performed. The studies represent the mean of at least 3 independent experiments. P values were determined comparing cells treated with DMH2 and SB alone to cells treated with both inhibitors. (TIF 2411 kb) [file 12943_2016_511_MOESM6_ESM.tif]
